# Beneath the Drought: Rhizosphere Processes Shape Genotype‐Specific Sorghum Responses to Drought‐Induced N Limitation

**DOI:** 10.1111/pce.70845

**Published:** 2026-08-31

**Authors:** Rosepiah Munene, Osman Mustafa, Sara Loftus, Vincent O. Suba, Nipuna Withanage, Niklas Schmücker, Peter Bulli, Reimund P. Rötter, Dennis O. Otieno, Mutez A. Ahmed, Michaela A. Dippold

**Affiliations:** ^1^ Geo‐Biosphere Interactions, Department of Geosciences University of Tuebingen Tuebingen Germany; ^2^ Biogeochemistry of Agroecosystems, Department of Crop Sciences University of Goettingen Goettingen Germany; ^3^ Institute of Bio‐ and Geosciences IBG‐3: Agrosphere Juelich Research Centre Juelich Germany; ^4^ Department of Botany and Agricultural Biotechnology, Faculty of Agriculture University of Khartoum Khartoum North Sudan; ^5^ Guangdong Provincial Key Laboratory of Applied Botany, South China Botanical Gardens Chinese Academy of Sciences Guangzhou China; ^6^ National Ecological Science Data Centre Guangdong Branch, South China Botanical Garden Chinese Academy of Sciences, Chinese Academy of Sciences Guangzhou China; ^7^ School of Agricultural, Forest and Food Science (HAFL) Bern University of Applied Sciences (BFH) Zollikofen Switzerland; ^8^ ETH Zürich, Department of Environmental Systems Science Zürich Switzerland; ^9^ Department of Plant, Animal and Food Sciences Jaramogi Oginga Odinga University of Science and Technology Bondo Kenya; ^10^ Tropical Plant Production and Agricultural Systems Modelling (TROPAGS) University of Goettingen Goettingen Germany; ^11^ Centre for Biodiversity and Sustainable Land Use (CBL) University of Goettingen Goettingen Germany; ^12^ Department of Biological Sciences Jaramogi Oginga Odinga University of Science and Technology Bondo Kenya; ^13^ Cluster of Excellence (EXC 3121): TERRA – Terrestrial Geo‐Biosphere Interactions in a Changing World University of Tübingen Germany; ^14^ Root‐Soil Interaction, School of Life Sciences Technical University of Munich Freising Germany

**Keywords:** arbuscular mycorrhizal fungi, drought, enzymatic activity, microbial biomass, nitrogen acquisition, sorghum genotypes

## Abstract

Droughts increasingly threaten crop productivity in nutrient‐depleted tropical soils. We investigated how water limitation influences rhizomicrobial traits (microbial biomass and functional community composition, enzyme activities) and nitrogen (N) uptake in three sorghum genotypes Makueni local (Mkl), Gadam (Gd), and IESH 22012 (IESH) under well‐watered and drought conditions. Depth‐specific (0–30 and 30–60 cm) ^15^N labelling traced N uptake at flowering and grain‐filling. Drought reduced grain N content across all genotypes but shoot N only in IESH. Gd enhanced ^15^N recovery in grains via post‐anthesis uptake from both depths under drought, whereas Mkl and IESH rather reallocated N within the plants towards the grain. Compared to well‐watered conditions, rhizosphere chitinase activity declined in Mkl under drought, while leucine aminopeptidase (LAP) activity remained unchanged. Under drought, microbial biomass decreased at flowering but recovered at grain‐filling, coinciding with enhanced arbuscular mycorrhiza fungi (AMF) colonisation. At grain‐filling, AMF associations with specific bacterial taxa aligned with grain N recovery, N‐utilisation efficiency, and LAP activity, displaying complementary roles within the plant‐AMF‐bacterial functional consortia in sustaining N acquisition. Overall, N acquisition in sorghum shifted in a genotype‐specific manner from reliance on microbial activity under well‐watered conditions to selective AMF‐bacterial partnership at grain‐filling under drought. These responses highlight the significant role of rhizosphere functional dynamics in sorghum N nutrition during reproductive stages under concomitant drought and nutrient limitation.

## Summary statement

Drought revealed contrasting nitrogen acquisition strategies among sorghum genotypes, from pre‐anthesis N remobilisation to AMF‐associated post‐anthesis N uptake, sustaining grain N supply and productivity under water limitation.

## 1 Introduction

In many tropical soils across Sub‐Saharan Africa, nitrogen (N) is one of the most limiting nutrients for crop production (Dimkpa et al. 2023; Kihara et al. 2020). Climate change exacerbates this problem through increasingly frequent and intense droughts, which not only reduce water availability but may also impair plant N uptake. These combined stresses can create a vicious cycle that severely constrains attainable yields, especially for smallholder farming systems dependent on rainfed agriculture (FAO 2023; Gebrehiwot and Gebrewahid 2016). Moreover, the strongly weathered and physically constrained characteristics of many tropical soils, such as Plinthosols, can further restrict N acquisition, as hardpan formation at relatively shallow depths impedes root penetration, further reducing nutrient availability from deeper soil layers (Ikazaki et al. 2018; Eze et al. 2014).

Sorghum (*Sorghum bicolour* L. Moench) is a drought‐tolerant C_4_ cereal crop widely cultivated across Sub‐Saharan Africa, contributing significantly to regional food security (Khalifa and Eltahir 2023; Ganeme et al. 2022). Its tolerance to abiotic stresses, particularly drought (Valiaparambil Sunoj et al. 2017), enables it to withstand harsh environmental conditions. However, sorghum is highly vulnerable to water deficits during the reproductive phase (flowering and grain‐filling), which may result in substantial yield losses (Abreha et al. 2021; Souza et al. 2021). These stages also represent critical windows for N uptake, which supports grain set, development, and ultimately, yield stability (Maltese et al. 2021; Zhang et al. 2021). Hence, understanding how sorghum manages its N nutrition under drought during flowering and grain‐filling is essential for improving productivity of this key food crop in dryland systems.

Rhizosphere processes play a critical role in nutrient acquisition, especially under stress conditions (Adeleke et al. 2024; Angulo et al. 2022). The rhizosphere harbours a dynamic microbial community that drives N cycling by enzymatic breakdown, mineralisation and symbiotic transfer of N to plants (Park et al. 2023; Pantigoso et al. 2022). Although drought stress often reduces microbial biomass and activity, plant‐microbe associations have been shown to mitigate effects of stress by enhancing nutrient availability (Sharma et al. 2025: Bastías et al. 2022). Among these microbes, arbuscular mycorrhiza fungi (AMF) form symbiotic relationships with the roots of many crops (Gómez‐Leyva et al. 2023; Saha et al. 2022). In return for organic carbon (C), AMF extend the absorptive capacity of plant roots, enabling access to nutrient pools beyond the immediate rhizosphere volume, an important function, particularly under drought conditions when water mass flow and water films are interrupted (Abdalla et al. 2023; Wang et al. 2021).

Previous studies report that AMF can enhance N use efficiency (NUE), defined as yield per unit of N, by improving both uptake and utilisation, with varying effects across the plant's developmental stages (Basiru et al. 2025; Tang et al. 2022). Their contribution to N uptake tends to be lower during the vegetative phase than after flowering (Yang et al. 2022). In wheat, AMF colonisation has been shown to enhance post‐anthesis N uptake (Pellegrino et al. 2015), likely supporting grain development even as roots begin to senesce (Bogard et al. 2010). Under drought, the role of AMF in supporting N utilisation appears especially critical during grain‐filling (Xue et al. 2024; Fang et al. 2023; Yang et al. 2022). As most existing work has focused on AMF effects before flowering or under controlled conditions, it remains unclear whether sorghum maintains active AMF associations and functional benefits during the reproductive stages, particularly under drought stress. Addressing this gap is essential to decide whether promotion of this trait may counteract the increasing risk of drought‐induced N deficiency under future climatic condition.

Certain rhizosphere enzymes such as chitinase and leucine aminopeptidase (LAP) contribute to N cycling by mobilising organic N. While drought stress may suppress extracellular enzyme activity (Bogati and Walczak 2022; Staszel et al. 2022), responses can vary depending on enzyme and genotype (Qu et al. 2023). For instance, in sorghum, drought enhanced LAP activity but suppressed chitinase during the vegetative stage (Munene et al. 2025). To our knowledge, the impacts of drought on activities of rhizosphere key N‐cycling enzymes during reproductive stages, when N demand for grain development is critical and plants are particularly sensitive to stress, are not well documented, especially among different sorghum genotypes.

To investigate how water limitation affects rhizosphere microbial biomass, enzyme activities, and their subsequent impact on N nutrition during flowering and grain‐filling stages, we conducted a drought simulation field experiment in Siaya, Kenya, using rain‐out shelters combined with ^15^N isotope labelling. A factorial arrangement in randomised complete (RCBD) design was implemented with two water regimes (well‐watered and drought), two soil depths (0–30 cm and 30–60 cm), and three sorghum genotypes: Makueni Local (Mkl, landrace), Gadam (Gd, open‐pollinated variety), and IESH 22012 (IESH, commercial hybrid), all adapted to semi‐arid conditions. Soil and plant samples were collected at both phenological stages to quantify ^15^N‐labelled N uptake, soil microbial biomass, and the activities of key N‐cycling enzymes (chitinase and LAP). This study addressed three main questions: 1) How does drought affect N uptake and allocation among three different sorghum genotypes? 2) How do drought and plant developmental stages influence rhizosphere microbial communities, enzyme‐mediated N cycling (chitinase and LAP), and the functional interactions among bacteria, fungi, and AMF? 3) How do AMF associations and their interactions with microbial partners mediate N acquisition and allocation to sorghum under drought, and how is this symbiotic network shaped by plant genotype? Based on these questions, we tested three hypotheses: H1) drought reduces plant N uptake and alters N allocation patterns in sorghum, with these responses varying among genotypes, H2) drought modifies rhizosphere microbial activity and N‐cycling enzyme activities (chitinase and LAP), with effects differing across plant developmental stages and genotypes, and H3) AMF symbiosis positively influences plant N uptake, with this effect differing between water regimes and genotypes.

## 2 Materials and Methods

### 2.1 Study Site

The field experiment was conducted at the Siaya Agricultural Campus field station of Jaramogi Oginga, Odinga University of Science and Technology (JOOUST), Siaya, Kenya (0°5'39″ N, 34°28'52.1″ E).The study site has a semi‐arid tropical climate with a bimodal rainfall pattern. The long rains (March–May) receive 480–600 mm, while the short rains (October‐December) receive 300–400 mm, with an average annual temperature of 23°C at an elevation of 1310 m above sea level. The soil is classified as haplic Plinthosols, characterised by iron and manganese concretions forming a sub‐surface hardpan typically occurring at ~30–50 cm depth (IUSS Working Group WRB 2015). The texture is classified as sandy loam (54.7% sand, 12.7% silt, and 32% clay) with a bulk density of 1.57 g cm^−3^, soil water holding capacity at field capacity of 28.15%, pH (H_2_O) of 6.02, total organic carbon of 0.78%, and total N of 0.08%. The soil is generally well‐drained but has too low fertility and limited moisture retention capacity (Garcia et al. 2013).

### 2.2 Experimental Set‐up

Three sorghum genotypes; Mkl, Gd, and IESH were obtained from the International Crops Research Institute for the Semi‐Arid Tropics (ICRISAT), Kenya. These genotypes represent a gradient of breeding intervention and genetic improvement, comprising a landrace (Mkl), an open‐pollinated variety (Gd), and a hybrid (IESH), as described by Munene et al. (2025). They were selected for their contrasting breeding histories, providing an ideal basis for evaluating how genetic background influences plant and rhizosphere responses to water stress. The experiment included two water regimes: well‐watered and drought. A total of 24 plots (6 × 6 m each) were arranged in a randomised complete block design with four replications. Seeds were directly sown at an inter‐row spacing of 0.75 m and intra‐row spacing of 0.20 m. Each subplot contained seven rows, except the two outermost subplots, which had eight rows to minimise edge effects. Thinning and gapping (replacing missing plants) were done 15 days after seedling emergence to achieve uniform plant stands. Weeds were manually removed at the 3–4 leaf stage and again at the 6–8 leaf stage.

### 2.3 Installation of Rain‐Out Shelters

Drought conditions were simulated by use of metal‐framed interrow rain‐out shelters covered with polythene foil. Each shelter (6.0 × 0.55 m base area) was positioned between sorghum rows to intercept rainfall, allowing only minimal water input through uncovered gaps and stemflow (Figure 1a). The shelters were designed in a V‐shape for efficient rainwater collection and drainage, elevated 0.30 m above ground, and slanted at 5° to ensure smooth and rapid runoff. Installation was carried out at the 7–9 leaf stage, about 18 days before 50% flowering for Gd and IESH, and roughly 1 month before 50% flowering for Mkl. Soil moisture content (SMC) was continuously monitored throughout the season using TEROS 10 sensors (METRE Group Inc., Munich, Germany), installed in one representative replicate at exactly 15 cm and 45 cm depths in both water regimes prior to sowing. The remaining replicates were monitored using SoilWatch sensors calibrated against the TEROS 10 measurements. Sensors recorded SMC hourly, and daily averages were calculated.

Rainfall exclusion efficiency of the shelters was estimated by comparing changes in SMC (ΔSMC) following two contrasting rainfall events (7.3 and 36.2 mm) during the sheltering period. Because rainfall interception occurred at the soil surface, calculations were based on changes in SMC in the 0‐30 cm layer, which directly reflects differences in incoming precipitation. Changes in SMC were converted to equivalent water depth (mm water = ΔSMC × soil depth in mm) and compared between sheltered and well‐watered plots. Across both rainfall events, the increase in soil water storage in the sheltered plots was approximately 25% of that observed in well‐watered plots, corresponding to approximately 75% exclusion of incident rainfall. The remaining rainfall contribution was attributed to water entering through uncovered inter‐row areas and stemflow.

### 2.4 ^15^N Deep Labelling

To evaluate the effect of drought on N uptake from different soil depths, a stable isotope tracer, ^15^N as ammonium (^15^NH_4_^+^) and nitrate (^15^NO_3_^−^) ions (Sigma‐Aldrich, Germany), was applied using a deep‐labelling approach (Stock et al. 2022). One plant per plot and per depth was selected for labelling. Tracer application was carried out with a tube‐in‐a‐pipe system approach, consisting of a flexible Teflon tube inserted into a rigid PVC guide pipe for precise depth placement. The tube end was sealed and perforated along the last 10 cm to ensure uniform tracer release to all 360°. A 40 mL syringe attached to the injector tube was used for ^15^N tracer application, with separate syringes designated for each soil. Prior to injection, four evenly spaced holes were drilled around each selected plant to ensure uniform distribution of the tracer within the root zone. Each plant received four injections (20 mL per injection) at the target depths of 15 cm for the 0–30 cm layer and 45 cm for the 30–60 cm layer. These depths were chosen to allow adequate percolation within each soil layer. The ^15^N tracer was applied at a total rate of 0.4 mmol N per plant at 15 cm and 0.799 mmol N per plant at 45 cm, supplied as a mixture of ^15^NH_4_^+^ and ^15^NO_3_^−^. Labelling was performed at two phenological stages: 50% flowering (BBCH 65) and grain‐filling (BBCH 71‐73).

### 2.5 Plant Harvesting, Root Depth Distribution Assessment and Soil Sampling

Six days after ^15^N labelling, plants were destructively harvested, with shoots and roots separated. Loosely adhering soil was gently shaken off, and rhizosphere soil for phospholipid fatty acid (PLFA) analysis was collected, while fine lateral roots from node 3–5 were selected for AMF colonisation analysis. Both rhizosphere soil and root samples were stored in a cool box during fieldwork, and subsequently frozen at −20°C until analysis. Shoots were oven‐dried at 60°C for 12 h, whereas grain samples were air‐dried to a constant weight before dry weight was recorded. Soil profile trenches were excavated in each plot in front of two plant rows to access undisturbed soil profiles. Fresh rhizosphere soil samples were collected from the exposed profile face using a clean spatula at two depths (0–30 cm and 30–60 cm). Profiles were re‐excavated immediately before sampling at each time point to ensure collection of fresh soil for enzyme activity analyses. Root distribution was assessed on the same exposed soil profile face using a profile‐wall mapping method (Böhm 1979). A transparent polythene sheet (1.25 m wide × 1 m deep), spanning two adjacent plants, was pressed against the profile face, and all visible roots were traced in situ within the 0–30 and 30–60 cm depth increments. Root mapping was conducted at flowering and grain filling, coinciding with soil profile sampling.

### 2.6 Determination of Parameters

#### 2.6.1 ^15^N Isotope and N Contents Determination

Oven‐dried (60°C) samples were separately ground and homogenised. Subsamples were shipped to the University of Göttingen, Germany, where they were finely ball‐milled prior to analysis for total N and ^15^N enrichment. Measurements were conducted using an elemental analyser (Flash, ThermoFisher Scientific, Bremen, Germany) coupled to an isotope ratio mass spectrometer (IRMS) (Delta Plus Conflo III, ThermoFisher Scientific, Bremen, Germany). To calculate ^15^N recovery in plant material, the natural ^15^N abundance from unlabelled control plants was used as a baseline using equation 1. N stock (total N pool expressed as kg N ha^−1^), was calculated from measured N values and corresponding dry biomass for shoot and grains where applicable

(1) $${[}^{15}{\text{N}]}_{\text{acqui}\;}\;=\;{\left[\text{N}\right]}_{\text{lab}}*\;\frac{{\text{at}\%}^{15}{\text{N}}_{\text{lab}}{\;-\;\text{at}\%}^{15}{\text{N}}_{\text{nat}}}{{\text{at}\%}^{15}{\text{N}}_{\text{tracer}}{\;-\;\text{at}\%}^{15}{\text{N}}_{\text{nat}}},$$

where [^15^N] _acqui_ is the ^15^N amount taken up (^15^N in plant dry biomass [mg g^−1^]), [N]_lab_ is the total N content of the labelled plant material (N in total plant dry biomass [mg g^−1^]), at%^15^N_lab_ is the atom percent ^15^N of the labelled material while ^15^N_nat_ is the atom percent ^15^N of the non‐labelled plant material and ^15^N_tracer_ refers to the injected tracer (99 at%). ^15^N recovery was calculated as the proportion of applied ^15^N recovered in above‐ground biomass and grain. Each ^15^N dose per plant was divided by four to account for radial diffusion of the tracer from the four injection points, for tracer applications at 15 cm (0–30 cm) and 45 cm (30–60 cm) soil depths at both flowering and grain‐filling stages.

#### 2.6.2 Determination of N Remobilisation and Associated Indicators

Different parameters related to N dynamics and uptake were calculated, including N remobilisation efficiency (NRE), contribution of post‐anthesis N uptake (CANU), N utilisation efficiency (NU_t_E). NU_t_E was determined at flowering using aboveground biomass and at grain filling using both aboveground biomass and grain yield, while NRE and CANU were restricted to grain filling as they quantify post‐anthesis N dynamics. The calculations were done using the formulas below (Ru et al. 2024; Gaju et al. 2014; Gallais et al. 2007).

(2) $$\text{NRE}(\%)=\left(\frac{{\text{N}}_{\text{flowering}\;-\;}{\text{N}}_{\text{maturity}}}{{\text{N}}_{\text{flowering}}}\right)*100,$$

(3) CANU(%)=100−NRE,

(4)

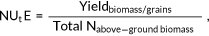

where N_flowering_ and N_maturity_ are the total N in the above ground biomass at flowering and at harvest (after removal of the grains) in kg^−1^ ha, respectively, and Yield _(biomass/grains)_ is the shoot biomass or grain weight at flowering and grain filling (kg ha^−1^) while Total N in the N in aboveground biomass (including grains) in kg N ha^−1^.

#### 2.6.3 Phospholipid Fatty Acid Analysis

Microbial biomass was determined using phospholipid fatty acid (PLFA) analysis, where PLFAs act as biomarkers for different functional microbial groups (Joergensen 2022; Zelles 1999). PLFA analysis was restricted to rhizosphere soil collected from the 0‐30 cm layer, as sampling sufficient rhizosphere soil from 30 to 60 cm was constrained by the hardened plinthic horizon, particularly under drought conditions. Extraction followed a modified protocol (Frostegård et al. 1991), as described by Dippold and Kuzyakov (2016). All chemicals used were of p.a. grade, obtained from Sigma‐Aldrich, Munich, Germany. Briefly, 10 g of frozen soil were extracted with methanol, chloroform, and citrate/KOH buffer (pH 4) in a 1:2:0.8 (vol/vol/vol) ratio. Before extraction, 25 μg of dinonadecanoylglycerol‐phosphatidylcholine (IS 1; 1 μg μL^−1^, Larodan, Sweden) were added as an internal standard to each sample. Phospholipids were separated from neutral and glycolipids by solid‐phase extraction (SPE) and subjected to basic esterification using 0.5 M NaOH in methanol at 100°C for 10 min. Methylation was then performed with BF_3_ (1.3 M)‐methanol at 80°C for 15 min to generate fatty acid methyl esters (FAMEs). FAMEs were extracted with hexane and dried under N_2_ gas. A second internal standard (IS 2; 15 μL of 1 μg μL^−1^ tridecanoic acid methyl ester in toluene) and 185 µL of toluene were added before transferring the samples into GC vials. Quantification and identification of FAMEs were performed by gas chromatography‐mass spectrometry (GC 7820 A, MS 5977B; Agilent Technologies, Waldbronn, Germany).

#### 2.6.4 Enzyme Assay

To assess drought effects on enzymatic activities, extracellular enzyme assays targeting chitinase and LAP were conducted. Soil samples were collected from two depths (0–30 cm and 30–60 cm) under well‐watered and drought conditions at flowering and grain‐filling stages. Soil closely adhering to roots was scraped with sterile spatulas, placed in sterile Eppendorf vials, and stored at 4°C until analysis. Activities of the extracellular enzymes were determined using synthetic fluorogenic substrates (German et al. 2011). Soil subsamples (1 g) were shaken with 50 m sterile water for 30 min and sonicated (40 J s^−1^, 2 min). Aliquots (50 μL) were transferred to black 96‐well microplates, each receiving 50 μL of buffer (10 µM MES for MUF‐linked; or TRIZMA, for AMC‐linked substrates) and 100 μL of substrate solution (0‐200 μmol g^−1^). Fluorescence was measured using a microplate reader (Victor^3^ 1420–050 Multilabel Counter, PerkinElmer, Waltham, MA, USA) at excitation and emission wavelengths of 355 and 460 nm, respectively. Enzyme activities were calculated from the regression slopes of standard curves and expressed as µmol substrate g^−1^ soil h^−1^. Activities were fitted to the Michaelis‐Menten equation (Equation 5) describing non‐linear substrate saturation kinetics.

(5) $$V=V_{\max}\;*\;S\;/\;K_{m}+S$$

where *V*_*max*_ is the maximum rate of enzymatic activity, S is the added substrate concentration, and *K*_*m*_ is the half‐saturation constant (substrate concentration at ½ *V*_*max*_) as indicator for substrate affinity.

#### 2.6.5 AMF Staining

Colonisation of AMF assessed using a modified ink‐staining protocol based on Vierheilig et al. (1998). Root samples were first bleached in 5% KOH at 95°C for 4 min, stained with Pelikan ink solution, and de‐stained before storage in glycerol at 4°C. Root segments were examined under a light microscope (Olympus BX40 fitted with Olympus CMOS‐camera SC50M) at 150x magnification (Olympus Ach 10x/0.25 ph∞ 0.17, ocular 15x) and 4.9 Mpx resolution (Olympus Europe SE & Co. KG, Hamburg, Germany). All major intraradical AMF structures (hyphae, arbuscules and vesicles) were visually identified. However, AMF colonisation was quantified only based on intraradical hyphae presence using gridline intersections and expressed as the percentage of colonised intersections across approximately 60 root segments per treatment.

### 2.7 Statistical Analyses

All statistical analyses were performed using R version 4.4.1 (R Core Team, 2024). Linear mixed‐effects models (LMM) were fitted using the lme4 package (Bates et al. 2015), with experimental blocks (four replicates; randomised complete block design) included as random effects to account for spatial heterogeneity in the field site. Water availability, genotype were treated as fixed effects and soil depth as an additional fixed factor where applicable to account for depth‐related differences. Where significant effects were detected, pairwise comparisons were performed separately for each depth. Growth stages (flowering and grain‐filling) were modelled independently as they represent physiologically distinct periods with differing water stress levels.

When assumptions of normality and homogeneity of variance were violated (as assessed by the Shapiro‐Wilk and Levene's tests, respectively), Box‐Cox transformation was applied to meet model assumptions. Two‐way analysis of variance (ANOVA) was used to analyse physiological parameters, N content and concentration, N use indices (NRE, CANU, NU_t_E), and PLFA profiles, with water availability and genotype as fixed factors. A three‐way ANOVA was conducted for ^15^N uptake and enzyme kinetic parameters (V_max_ and K_m_), including water availability, genotype, and soil depth as factors. Post‐hoc comparisons were performed using Tukey's HSD test at a significance level of α < 0.05.

Microbial groups were classified based on factor analysis of PLFA profiles, following their loading values and direction of association (Supporting Information Table S1) according to (Zelles 1997)) and Ba et al. 2022). Gram‐negative bacteria were separated into two groups: G‐1 (16:1ω7c), G‐2 (18:1ω7c). Gram‐positive groups included G + 1 (i15:0, i16:0), G + 2 (a15:0, i17:0), and G + 3 (a17:0). Actinobacteria (Actb) were grouped as Actb 1 (10Me16:0), Actb 2 (10Me17:0), and Actb 3 (10Me18:0). Fungal markers included 18:2ω6,9 and 16:1ω5c, as indicators of saprophytic and AMF respectively, while cy17:0 and cy19:0 were used as stress indicators (Joergensen 2022; Willers et al. 2015).

Non‐metric multidimensional scaling (NMDS) was used to visualise clustering patterns in functional microbial community composition across treatments (Ramette 2007). Bray‐Curtis dissimilarities were calculated using vegdist function in the *vegan* package and plotted using metaMDS function. Permutational multivariate analysis of variance (PERMANOVA) was used to test the effects of drought and genotype using the adonis2 function, also based on Bray‐Curtis distances. Redundancy analysis (RDA) was performed with the *vegan* package to evaluate the relationship between composition of functional microbial groups, enzymatic activities and N use dynamics (NRE, CANU, NU_t_E) under different water regimes. The data were log‐transformed and scaled prior to RDA analysis, and Type II scaling (correlation) plots are presented. The traced polythene sheets were digitally scanned, and root marks were quantified using Claude (Anthropic), an AI‐assisted image analysis tool. Root counts were recorded for the 0–30 and 30–60 cm layers and used as a quantitative measure of root distribution.

## 3 Results

### 3.1 N Content and Mycorrhizal Colonisation

Drought significantly reduced shoot N stock/amount per ha for IESH at both flowering and grain‐filling, while Mkl and Gd remained unaffected at both stages (Figure 1b–c). Grain N amount per ha declined significantly for all genotypes under drought (Figure 1d). Shoot N content was not affected by drought at both flowering and grain‐filling but showed significant genotypic differences (Figure 1e–f), suggesting that the reduction in plant N stock largely reflected reduced biomass accumulation rather than changes in tissue N content. Gd exhibited higher shoot N content than Mkl and IESH under drought and then Mkl under well‐watered conditions.

**Figure 1:**
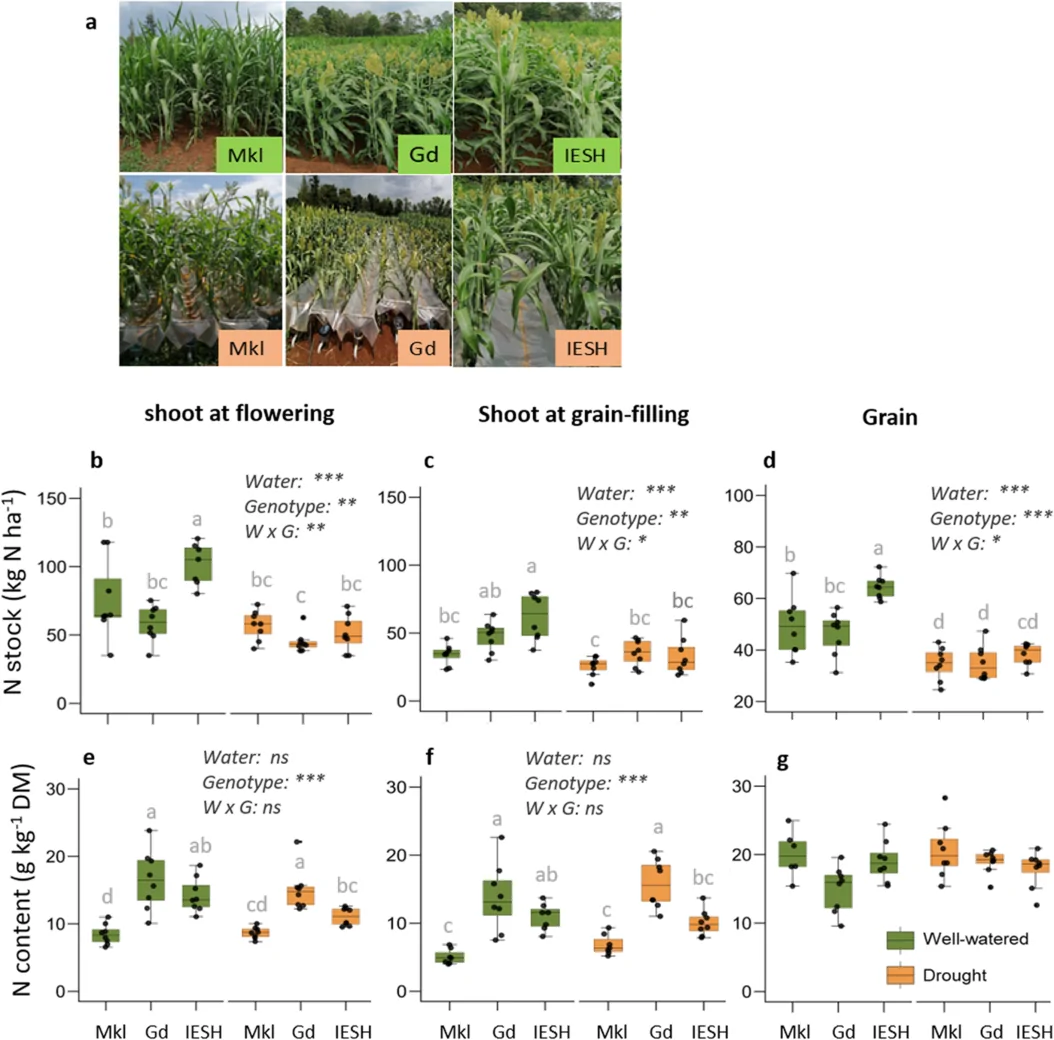
Nitrogen accumulation under contrasting water regimes. (a) Field plots without rain‐out shelters (well‐watered, top) and with rain‐out shelters (drought, below) conditions. Shoot N stock at flowering (b) and at grain‐filling (c), grain N stock (d), shoot N content at flowering (e), and at grain‐filling (f), and grain N content (g) in sorghum genotypes under well‐watered (green) and drought (orange) conditions. Sorghum genotypes: Mkl (Makueni local), Gd (Gadam), and IESH 22012. Boxplots show median, interquartile range and outliers. Different letters indicate significant differences (*p* < 0.05) between treatments (water availability and genotype) identified by LMM. ***p < 0.001, **p < 0.01, *p < 0.05 and ns, not significant.

In contrast, grain N content remained stable across water treatments and genotypes, showing no significant differences (Figure 1g). At flowering, water availability had no significant effect on AMF colonisation, whereas genotype showed a weak but significant main effect (*p* = 0.04), with no clear pairwise differences among genotypes after post‐hoc adjustment (Figure 2a). By grain filling, however, drought significantly increased AMF colonisation (*p* < 0.05), with Gd exhibiting greater colonisation than Mkl under drought conditions (Figure 2b).

**Figure 2:**
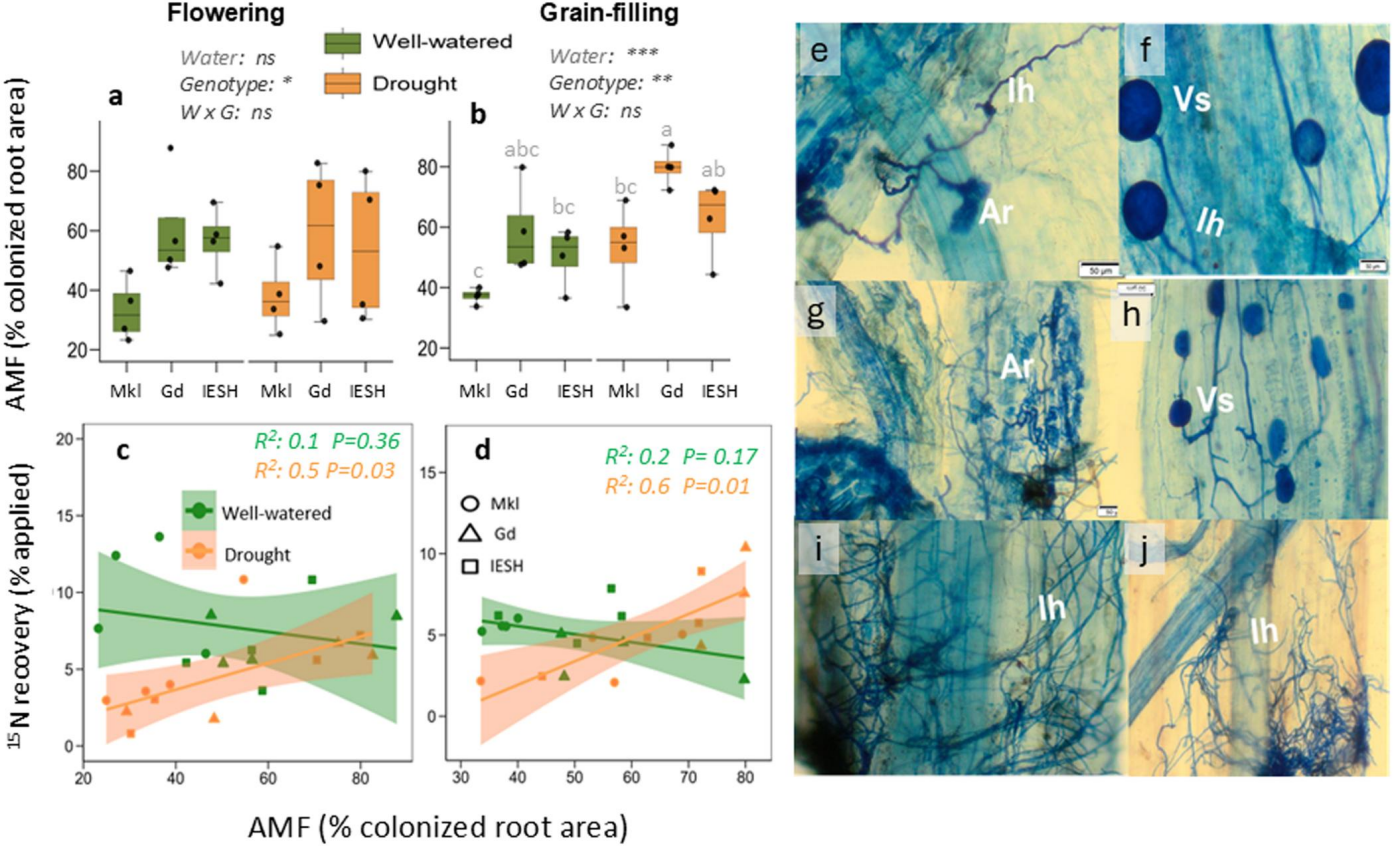
Percentage of AMF root colonisation on sorghum genotypes at flowering (a) and grain‐filling (b) and their correlations ^15^N recovery in the shoot biomass (c) and in the grains (d) under well‐watered (green) and drought conditions (orange). Photomicrographs (e–j) of sorghum root colonisation by AMF, illustrating structural components: intraradical hyphae (Ih), arbuscules (Ar), and vesicles (Vs). Images taken at ×150; scale bar = 50 µm. The sorghum genotypes Mkl (Makueni local; circle), Gd (Gadam; triangle), and IESH (IESH 22012; square). Boxplots show median, interquartile range and outliers. Different letters indicate significant differences (*p* < 0.05) between treatments (water availability and genotype) identified by LMM. *p*‐values of ANOVA of water availability (W), genotype (G) and their interactions are indicated as ***p < 0.001, **p < 0.01, *p < 0.05 and ns, not significant. [Color figure can be viewed at wileyonlinelibrary.com]

### 3.2 ^15^N Uptake, N Utilisation and Remobilisation Dynamics

Interestingly, drought did not significantly alter ^15^N uptake from both depths in shoot biomass for all genotypes at flowering stage compared to well‐watered conditions (Figure 3a). At grain‐filling stage, however, drought significantly (*p* < 0.001) reduced ^15^N uptake from the upper depth (0–30 cm) into shoot biomass for Mkl, with no significant changes for Gd and IESH genotypes (Figure 3b).

**Figure 3:**
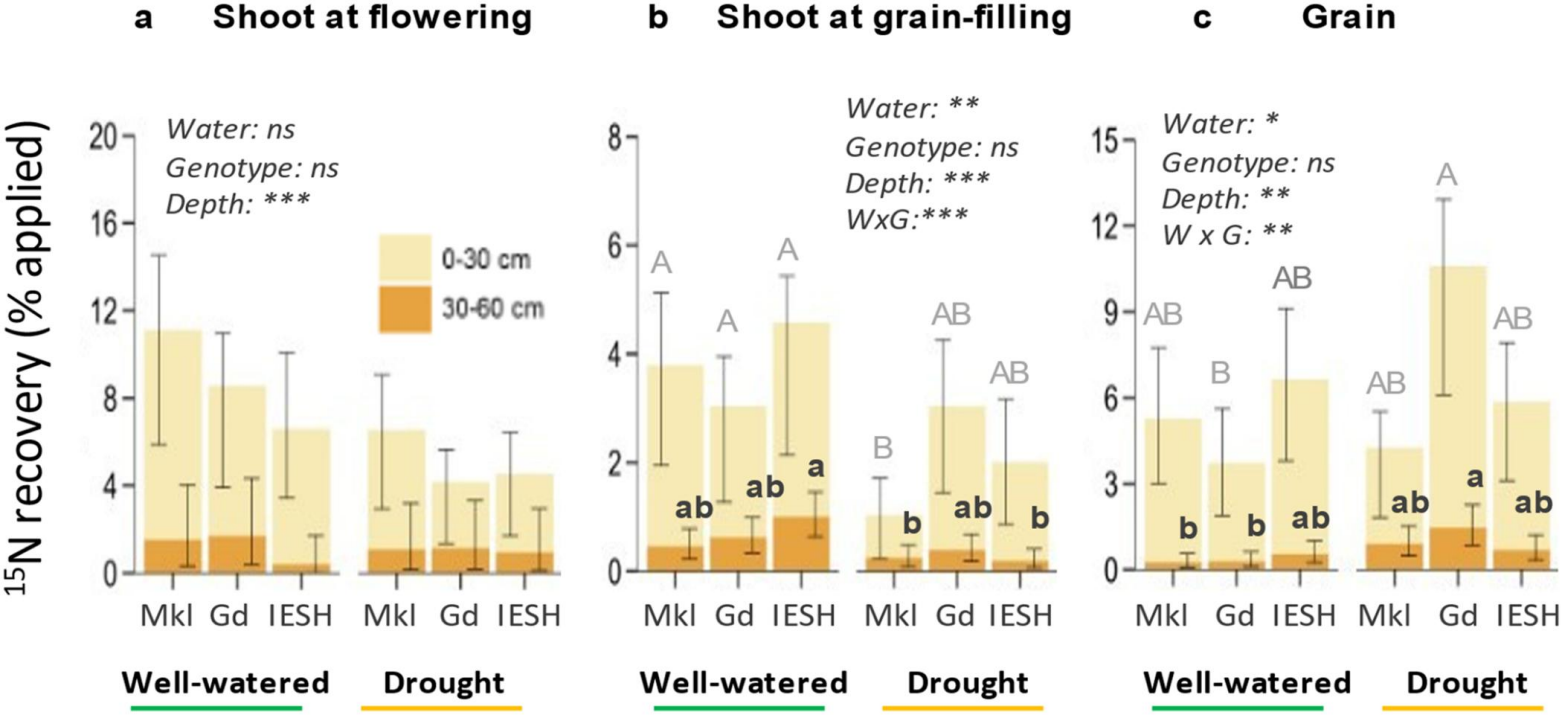
Proportion of the applied ^15^N recovered in the shoot biomass at flowering (a), at grain‐filling (b), and in grains (c) under well‐watered (left) and drought conditions (right). The ^15^N tracer was applied at two different soil depths: 0–30 cm (light brown bar on top) and 30–60 cm (brown bar at bottom). The sorghum genotypes are denoted as Mkl (Makueni local), Gd (Gadam), and IESH (IESH 22012). Different letters indicate significant differences (*p* < 0.05) between treatments (water availability and genotype) with respect to ^15^N recovery from 0 to 30 cm (small letters) and 30‐60 cm (capital letters), respectively. *p*‐values of ANOVA of water availability (W), genotype (G) and their interaction as well as of soil depth are indicated as ***p < 0.001, **p < 0.01, *p < 0.05 and ns, not significant. [Color figure can be viewed at wileyonlinelibrary.com]

From the lower soil layer (30‐60 cm), ^15^N uptake into the shoot was significantly reduced under drought for IESH, but not for Gd or Mkl (Figure 3b). While drought did not significantly affect the recovery of ^15^N in the grains for Mkl and IESH, recovery of the applied mineral ^15^N in Gd grains surprisingly exhibited a 3‐ and eightfold increase from the upper and lower depths, respectively (Figure 3c). This is interlinked to the observation that under drought conditions, AMF colonisation showed a significant correlation with ^15^N recovery both at flowering and grain‐filling (Figure 2c–d). Specifically, while the relationship was dependent on water availability, the AMF x water interaction was not significant at flowering but was highly significant at grain filling (*p* < 0.001), indicating that water treatment influenced the AMF‐ ^15^N relationship. Additionally, the AMF x genotype interaction was marginal at flowering (*p* = 0.08) and significant at grain filling (*p* = 0.01), suggesting that genotype‐specific differences in the AMF‐^15^N relationship became more pronounced during grain filling under drought. In addition, at flowering, NU_t_E_biomass_ was significantly influenced by genotype (*p* < 0.001), with Gd being notably lower compared to Mkl and IESH under drought conditions (Table 1). At the grain‐filling stage, drought reduced NU_t_Ebiomass only in Mkl by 28% compared to well‐watered conditions, whereas Gd and IESH were unaffected by water limitation.

**Table 1:** Nitrogen use efficiency traits in sorghum under well‐watered and drought conditions. N utilisation efficiency (NU_t_E) at flowering, N remobilisation efficiency (NRE), contribution of post‐anthesis N uptake (CANU) and N utilisation efficiency biomass (NU_t_E_biomass_)/grain‐based (NU_t_E_grain_) at grain‐filling in sorghum genotypes under well‐watered and drought conditions.

Water‐availability	Genotype	Flowering	Grain‐filling			
		NU_t_E_biomass_ (kg DW kg^−1^ N)	NRE (%)	CANU (%)	NU_t_E_grain_ (kg DW kg^−1^ N)	NU_t_E_biomass_ (kg DW kg^−1^ N)
Well‐watered	Mkl	121.9 ± 5.5 a	52.6 ± 3.2 ab	42.4 ± 5.4 b	32.4 ± 4.2 b	85.6 ± 10.1 a
	Gd	55.4 ± 7.8 c	31.2 ± 4.2 b	69.0 ± 4.2 a	43.1 ± 1.6 ab	42.3 ± 11.0 c
	IESH	74.0 ± 4.8 bc	51.2 ± 6.0 ab	48.8 ± 6.0 ab	33.7 ± 1.8 b	42.8 ± 6.3 c
Drought	Mkl	116.2.1 ± 4.7 a	54.3 ± 6.4 a	45.7 ± 6.4 b	27.9 ± 4.2 b	61.9 ± 14.5 bc
	Gd	56.5 ± 6.3 c	32.3 ± 2.8 b	67.7 ± 2.8 a	35.7 ± 4.7 ab	32.6 ± 3.3 c
	IESH	91.0 ± 5.1 b	39.6 ± 3.0 ab	60.4 ± 2.9 ab	51.4 ± 4.90 a	47.4 ± 7.1 c
**Statistical significance**						
Water		ns	ns	ns	ns	*
Genotype		**	**	**	**	***
Water x Genotype		*	ns	ns	**	**

*Note:* Sorghum genotypes: Mkl (Makueni local), Gd (Gadam), and IESH (IESH 22012). Data are presented as mean ± SE. Different letters indicate significant differences (*p* < 0.05) between treatments (water availability and genotypes) identified by LMER. p‐values of ANOVA of water availability, genotype and their interactions are indicated as ***p < 0.001; **p < 0.01; *p < 0.05 and ns, not significant.

Moreover, NRE and CANU were influenced by genotype but not by water availability. Mkl showed a higher NRE than Gd under drought conditions, whereas Gd exhibited greater CANU than Mkl under well‐watered and drought conditions. For IESH neither NRE nor CANU was significantly affected by water availability.

### 3.3 Soil Enzymatic Activities

At flowering, water availability alone did not significantly affect chitinase V_max_; but a significant water × genotype interaction (*p* < 0.05) indicated genotype‐specific drought responses (Figure 4a). During grain‐filling, water, genotype and their interactions as well as soil depth significantly (*p* < 0.05) affected chitinase potential activities (Figure 4b). Specifically, at 0–30 cm depth, drought stress reduced the chitinase V_max_ values for Mkl by ~32% at flowering and ~38% at grain‐filling, whereas Gd and IESH remained unaffected (Figure 4a–b). At the lower depths (30–60 cm), V_max_ did not significantly differ between water treatments for all genotypes at either growth stages (Figure 4a–b). Unlike chitinase, LAP activities remained unaffected by drought across all the genotypes at both depths and development stages (Figure 4c–d). At flowering, no significant differences in K_m_ values were observed between well‐watered and drought conditions for LAP, indicating that enzymatic affinity for substrates remained unaffected (Supporting Information Figure S2c). In contrast, drought reduced the K_m_ value of chitinase at both flowering and grain‐filling stages (Supporting Information Figure S2a–b), but for LAP only at grain‐filling (Supporting Information Figure S2d).

**Figure 4:**
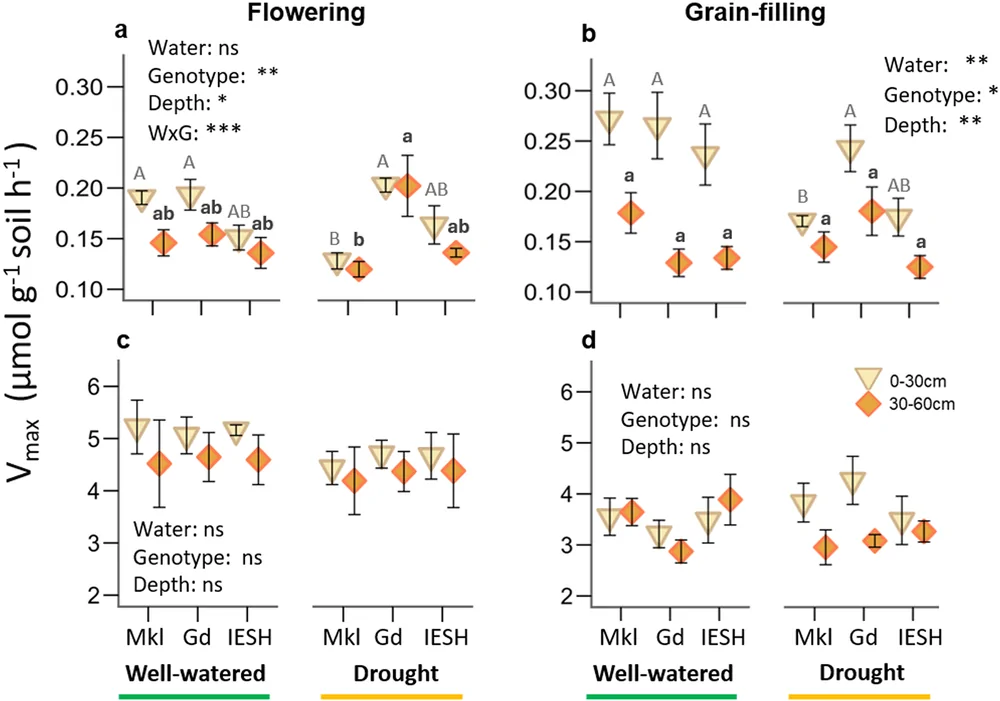
Potential enzyme activities (V_max_, µmol g^−1^ soil^−1^ h^−1^) of chitinase and leucine aminopeptidase LAP at flowering (a, c) and for both enzymes at grain‐filling (b, d), respectively. Well‐watered conditions are shown left and drought on the right. Soil depths are indicated by colour: 0–30 cm in light brown inverted triangles and 30‐60 cm in dark brown squares. The sorghum genotypes are Mkl (Makueni local), Gd (Gadam), and IESH 22012. Data are presented as means ± SE. Different letters indicate significant differences (*p* < 0.05) between sorghum genotypes and water availability on 0–30 cm (capital letters) and 30–60 cm (small letters), respectively. *p*‐values of ANOVA of water availability (W), genotype (G) and their interactions are indicated as ***p < 0.001, **p < 0.01, *p < 0.05 and ns, not significant. [Color figure can be viewed at wileyonlinelibrary.com]

### 3.4 Microbial Community Composition

The total PLFA content (ΣPLFA) per microgram rhizosphere soil, a proxy for microbial biomass, was significantly affected by water availability at flowering and grain‐filling stages (Table 2). At flowering, drought significantly (*p* < 0.05) decreased the total microbial biomass IESH, while that of Gd and Mkl was not significantly reduced. Correspondingly, the abundance of total bacterial PLFA also decreased in response to drought, with the IESH genotype showing a notable decrease of ~45%. This was driven by a significant drought‐induced decrease in the specific PLFA for G‐1, G‐2 and G + 2 (Supporting Information Figure S3). Additionally, under drought, actinobacterial markers (e.g., Actb2 and Actb3) fell below detection limit for IESH, and Actb2 for Gd, whereas Actb1 was not significantly affected by drought. In contrast, abundance of fungal PLFA was unaffected by drought across all genotypes (Table 2; Supporting Information Figure S3).

**Table 2:** Total microbial biomass PLFA abundance, bacterial, fungal, and stress indicators of Gram negatives at flowering and grain‐filling stages in sorghum genotypes under well‐watered and drought conditions.

PLFA (µg g^−1^soil)	Genotype	Flowering stage		Grain‐filling stage	
		Well‐watered	Drought	Well‐watered	Drought
Total microbial	Mkl	14.33 ± 1.29^a^	10.28 ± 0.55^ab^	9.82 ± 0.81^ab^	10.64 ± 0.60^a^
	Gd	12.98 ± 1.35^ab^	10.95 ± 0.69^ab^	8.90 ± 0.88^bc^	12.13 ± 1.06^a^
	IESH	13.18 ± 1.01^ab^	8.51 ± 0.33^b^	6.83 ± 0.83^c^	10.71 ± 0.93^ab^
∑ bacterial biomarkers	Mkl	12.13 ± 1.33^a^	8.11 ± 0.49^ab^	7.61 ± 0.87^abc^	8.75 ± 0.77^abc^
	Gd	10.05 ± 1.84^ab^	9.56 ± 0.93^ab^	7.07 ± 0.49^bc^	10.45 ± 0.98^a^
	IESH	11.12 ± 0.77^a^	6.09 ± 0.28^b^	5.16 ± 0.92^c^	8.78 ± 0.97^ab^
∑ fungal Biomarkers	Mkl	0.99 ± 0.05^a^	0.66 ± 0.06^a^	0.73 ± 0.03^a^	0.80 ± 0.11^a^
	Gd	1.05 ± 0.06^a^	0.82 ± 0.05^a^	0.70 ± 0.05^a^	0.88 ± 0.09^a^
	IESH	0.99 ± 0.07^a^	0.87 ± 0.06^a^	0.68 ± 0.02^a^	0.75 ± 0.09^a^
Fungal:bacterial ratio	Mkl	0.085 ± 0.01^a^	0.11 ± 0.004^a^	0.098 ± 0.01^ab^	0.090 ± 0.02^b^
	Gd	0.12 ± 0.03^a^	0.12 ± 0.01^a^	0.10 ± 0.01^ab^	0.084 ± 0.01^b^
	IESH	0.10 ± 0.01^a^	0.15 ± 0.02^a^	0.12 ± 0.2^a^	0.082 ± 0.2^b^
Stress ratio (Cy17:0/16:1ω7c)	Mkl	2.3 ± 0.8^b^	2.8 ± 0.5^b^	0.8 ± 0.9^a^	0.6 ± 0.08^a^
	Gd	3.4 ± 1.3^b^	4.1 ± 1.6^b^	0.8 ± 0.07^a^	0.7 ± 0.09^a^
	IESH	1.58 ± 0.6^b^	10.3 ± 2.3^a^	0.7 ± 0.04^a^	0.7 ± 0.05^a^
Stress ratio (Cy19:0/18:1ω7c)	Mkl	7.3 ± 1.5^bc^	7.3 ± 1.5^bc^	2.6 ± 0.2^a^	2.4 ± 0.4^a^
	Gd	6.7 ± 2.0^bc^	15.7 ± 3.6^a^	2.4 ± 0.3^a^	2.5 ± 0.3^a^
	IESH	4.3 ± 0.3^c^	15.4 ± 0.6a^b^	2.5 ± 0.2^a^	3.2 ± 0.6^a^

*Note:* Sorghum genotypes Mkl (Makueni local), Gd (Gadam), and IESH (IESH 22012. Data are presented mean ± SE. Different letters indicate significant differences (*p* < 0.05) between water and sorghum genotypes.

At the grain‐filling stage, drought had the opposite effect, with total microbial biomass increasing by 27% and 36% in the rhizosphere of Gd and IESH, respectively, compared to controls (Table 2). The abundance of the cyclopropyl fatty acid Cy17:0 was not affected by water availability, whereas Cy19:0 significantly increased under drought (Supporting Information Figure S3). Consequently, the Gram‐negative stress indicator ratios (Cy17:0/16:1ω7c and Cy19:0/18:1ω9c) were significantly elevated under drought at flowering stage. Drought induced a marginal increase of the fungal‐to‐bacterial ratio at flowering but a significant decrease under at grain‐filling.

NMDS ordination and PERMANOVA revealed that at flowering, microbial community composition was significantly influenced by water availability (*p* = 0.01, *R* ^2^ = 0.27) and genotype (*p* = 0.05, *R* ^2^ = 0.20), with a stress value of 0.13 (Figure 5a). By grain‐filling, only the influence of water availability remained significant (*p* = 0.001, *R* ^2^ = 0.20; stress = 0.15; Figure 5b), indicating a persistent drought effect across sorghum genotypes.

**Figure 5:**
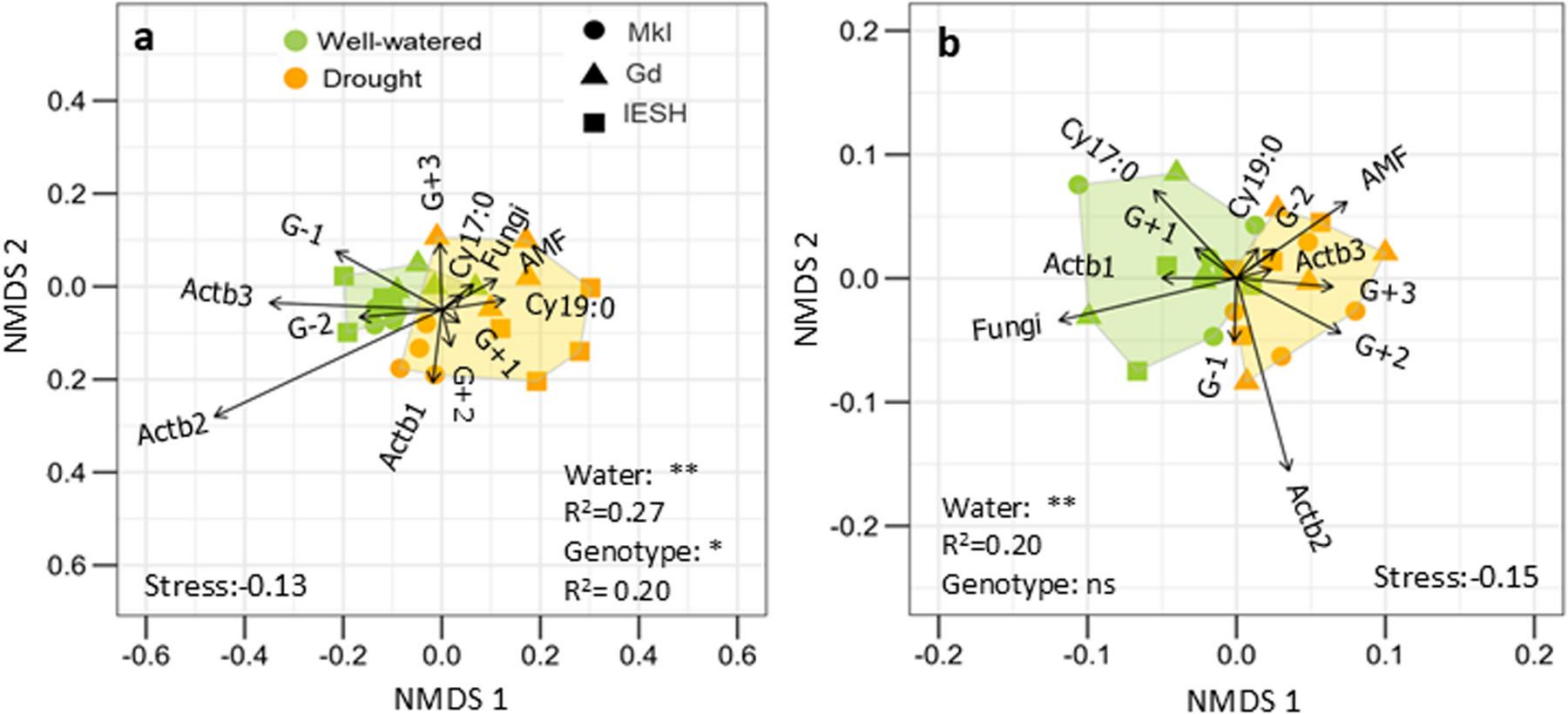
Non‐metric multidimensional scaling (NMDS) plots based on Bray‐Curtis dissimilarities showing the microbial community composition at flowering (a) and grain‐filling (b) under well‐watered (green) and drought (orange) conditions in sorghum genotypes. [Color figure can be viewed at wileyonlinelibrary.com]

### 3.5 Redundancy Analysis (RDA) of N Dynamics Under Drought and Optimal Conditions

The RDA revealed development/growth stage‐ and water‐specific relations of microbial associations with N‐related indices.

At flowering, under well‐watered conditions, ^15^N uptake, biomass N stock, and LAP activity were primarily associated with bacterial groups G + 2, G‐1 and G‐2 (Figure 6a). Under drought, NU_t_E showed a strong alignment with Actb1, whereas fungal markers (AMF and saprotrophic fungi) exhibited closer associations with the chitinase enzymatic activity (Figure 6a).

**Figure 6:**
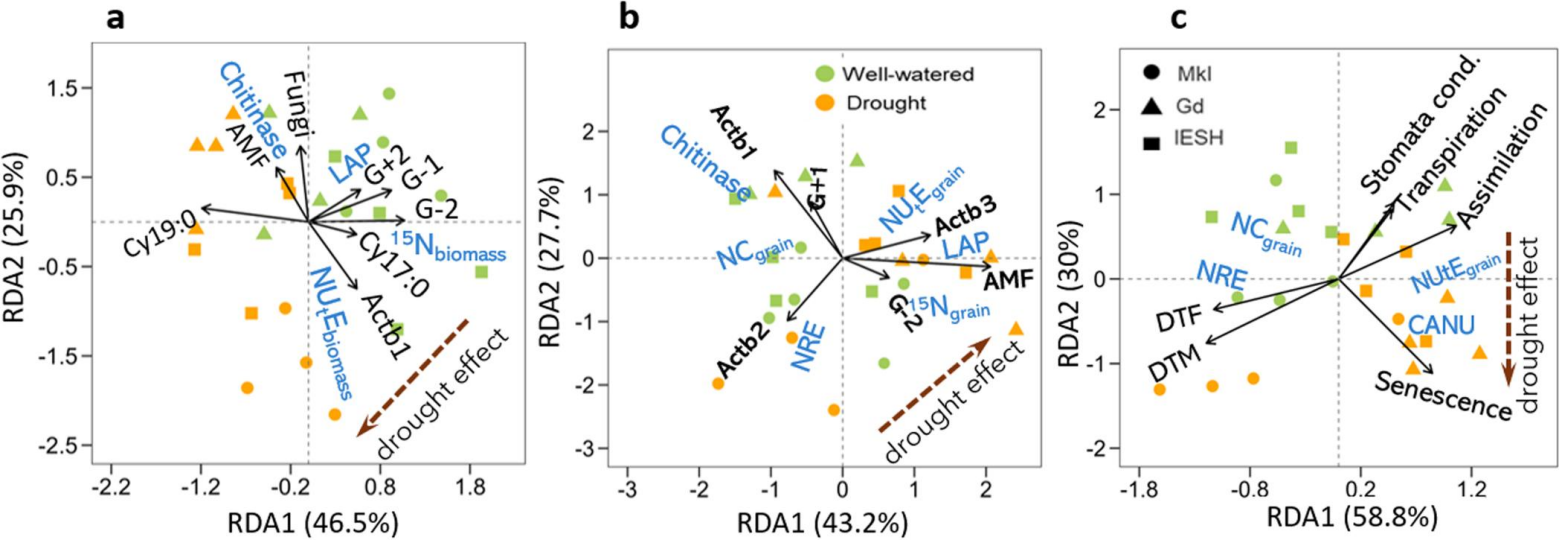
Redundancy analysis (RDA) triplots of N dynamics and microbial associations at flowering (a), and grain‐filling (b), and physiological attributes on N allocation in grains (c) under well‐watered (green) and drought (orange) conditions in sorghum genotypes (Makueni local; Mkl (circle), Gadam; Gd (triangle) and IESH 22012; IESH (square)). With microbial community as explanatory variables (black arrows) and enzymatic activity (chitinase, leucine‐aminopeptidase [LAP]), ^15^N_biomass_ (^15^N recovery in shoot biomass), ^15^N_grain_ (^15^N recovery in grains), NC_biomass_ (N stock/pool size in shoot biomass), NC_grain_ (N stock/pool size in grains), CANU (contribution of post‐anthesis N uptake), NRE (N remobilisation efficiency) and NU_t_E_biomass_ and NU_t_E_gr_ (N utilisation efficiency in biomass and in grain, respectively), DAT (days to flowering), DTM (days to maturity), as well as stomata conductance, transpiration and assimilation (Suba et al. 2025) as response variables. Type II scaling (correlation) plot is shown, with only significant constraining variables. The constraining variables explained 59% at flowering, 40% and 46% of the total variance at grain‐filling, respectively. [Color figure can be viewed at wileyonlinelibrary.com]

At grain‐filling, the pattern shifted. Under well‐watered conditions, G + 1 and Actb1 were closely correlated with V_max_ chitinase and grain N content (Figure 6b). Under drought, the AMF marker, G‐2, and Actb3 aligned in the same direction and were strongly associated with ^15^N recovery in grains, LAP activity, and contribution to post‐anthesis N uptake (CANU). They also showed a moderate association (cos^2^ = 0.68) with NU_t_E. The physiological attributes contributed strongly to explaining grain N allocation. Under well‐watered conditions, stomatal conductance and transpiration were closely associated with grain N content (Figure 6c). Extended maturity (DTF and DTM) also showed positive links with NRE more towards the drought, whereas senescence was more strongly associated with post‐CANU and NU_t_E aligned more closely with assimilation.

## 4 Discussion

### 4.1 Drought and Genotype Shape N Allocation in Sorghum

Drought reduces N uptake and plant growth (Supporting Information Figure S1) by disrupting transpiration‐driven mass flow of water to the roots (Suba et al. 2025; Gloser et al. 2020). Root system plasticity is a key determinant of N acquisition under drought in sorghum (Loftus et al. 2025). However, this study emphasises rhizosphere and rhizomicrobial processes as complementary drivers of genotype‐specific N uptake. Grain N content declined across all genotypes by drought (Figure 1c), indicating disrupted N acquisition and redistribution during reproductive growth. The landrace (Mkl) and open‐pollinated variety (Gd) appeared to maintain more conservative nutrient use under moisture limitation, whereas the hybrid (IESH), characterised by a more resource‐intensive growth strategy, was more sensitive to drought‐induced declines in N availability (Figure 1b–d). Genotypic differences in shoot N content reflect biomass dilution effects (Figure 1e–f), with Mkl's larger vegetative mass strongly diluting N contents despite stable N stock across the field, whereas Gd's lower biomass contributing to its consistently higher shoot N content (Suba et al. 2025; Zhang et al. 2022).

During grain‐filling, N accumulation depends on both post‐anthesis uptake and remobilisation of pre‐anthesis reserves (Ru et al. 2024; Sharma et al. 2023). The strong association between NRE and extended maturity, highlights the role of phenology for drought resilience. As a late‐maturing genotype, Mkl accumulated pre‐anthesis N that served as a ‘reserve’, exemplifying a reserve‐dependent strategy (Sharma et al. 2023; Mangin et al. 2022). Its high NRE allows efficient N cycling, as indicated by low shoot total N stock and reduced allocation of new, tracer‐derived N to shoot biomass at grain‐filling. In contrast, Gd relied more on a post‐anthesis acquisition strategy even under drought stress, as indicated by high ^15^N recovery in grains. Despite stable shoot N at both stages, grain total N content of Gd declined under drought, reflecting Gd's low NRE. Stress‐induced senescence likely reduces the efficiency of N re‐allocation, causing pre‐anthesis reserves to remain 'trapped' in vegetative tissues (Padhan et al. 2023; Sharma et al. 2023). In contrast, IESH had accumulated the high total N under well‐watered conditions at flowering, but this was substantially reduced under drought, indicating loss of its advantageous acquisitive traits in dry soil (Prey et al. 2019). Its high NU_t_E and stay‐green trait (Suba et al. 2025) could not fully compensate for the reduced pre‐anthesis N reserves under drought, resulting in significantly lower final grain N. Overall, drought influenced N allocation in sorghum in a genotype‐specific manner, but internal allocation alone was insufficient to sustain grain N, underscoring the importance of continued soil N uptake under drought.

### 4.2 Influence of Drought on N Uptake From Different Depths

At flowering, N uptake ‐ determined as ^15^N recovery ‐ was not significantly affected by drought at either soil depth, indicating stable N acquisition predominantly from topsoil (Figure 2d) which host higher root densities (Supporting Information Figure S7a) (Wang et al. 2024). Even after an extended drought at the grain‐filling stage, N acquisition occurred predominantly from the upper soil layer (0–30 cm). Although our previous pot experiment demonstrated greater drought‐induced root investment, particularly in Mkl, as indicated by an increased root/shoot ratio (Munene et al. 2025), this response did not necessarily translate into greater N uptake from deeper soil layers under field conditions (Jackson et al. 2008; Giehl and von Wiren 2014).

Under well‐watered conditions, unrestricted water mass flow to the roots ensured a high overall mobility of the total soil mineral N pool. In addition, strong tropical precipitation events also promote leaching of mineral N from the rooting zone, these phenomena together explain the high dilution of the applied ^15^N tracer, representing a reduced fraction of total plant N uptake (Lewicka‐Szczebak and Well 2020). In contrast, under drought conditions, reduced N mobility may have retained the tracer within the rooting zone, increasing the relative contribution of the applied tracer to plant N uptake, resulting in higher apparent ^15^N recovery despite overall lower N availability (Nasielski et al. 2019).

Gd exhibited substantially greater post‐anthesis recovery of labelled N under drought (Table 1), despite no corresponding increase in deep root distribution. Together with the significant positive association between AMF colonisation and topsoil‐derived ^15^N recovery and significant AMF × genotype interaction under drought at grain filling (Figure 2c–d). This suggests that genotype‐specific differences in the post‐anthesis N involved both rooting patterns and rhizosphere processes. However, because root distribution was assessed using profile‐wall mapping rather than quantitative measurements of root length density or biomass, these patterns should be interpreted as indicative of spatial rooting responses rather than direct estimates of plant investment to the root system or functional root activity.

### 4.3 Depth and Drought Effects on Soil Enzymatic Activity

Drought downregulated chitinase activity in Mkl at both flowering and grain‐filling stages in top (0‐30 cm) but not in the more moist subsoils (30–60 cm) (Figure 4a–b), underlying that the disruption in water films by topsoil drying causes reductions in enzyme and substrate diffusion (Stone et al. 2014; Ndossi et al. 2020). Additionally, subsoil extracellular enzymes, may bind to mineral surfaces, specifically in these iron‐rich soils, making their overall activity less sensitive to reduction of water film connectivity under drought (Nannipieri et al. 2018). The drought‐induced decline in topsoil chitinase activity occurred despite stable fungal biomass, indicating genotype‐specific regulation of microbial enzyme activity rather than changes in the abundance of the fungal chitinase producers.

Contrary to expectations, soil LAP activity remained stable under drought at both flowering and grain‐filling (Figure 4c–d), suggesting active microbial support of protein depolymerisation under water stress (Zhang et al. 2024). The sustained activity may reflect functional persistence of drought‐tolerant microbial taxa in the topsoil irrespective of a potentially reduced abundance, but may also reflect an increase root secretion of LAP (De Silva et al. 2025; Metze et al. 2023; Greenfield et al. 2021). The decrease in chitinase and LAP *K*_*m*_ values, particularly for Mkl (Supporting Information Figure S2), suggests an increase in substrate affinity. This likely reflects compensatory adjustments to the reduced substrate availability under drought stress, and indicates the expression of isoenzymes with higher substrate affinity (Tang and Riley 2019).

Collectively, these mechanisms largely sustain soil N mineralisation capacity under drought, thus, buffering sorghum against N limitation even under low topsoil moisture supply. The decline in the chitinase:LAP ratio under drought at the grain‐filling stage (Supporting Information Figure S3) suggests a shift in N acquisition from complex sources like chitin to more labile peptides and proteins (Scranton et al. 2012; Stock et al. 2019), most likely driven by bacteria, as indicated by a declining fungal:bacterial ratio under drought (Supporting Information Figure S3) (Wang and Kuzyakov 2024).

### 4.4 Dynamic Microbial Alliances Enhance Nutrient Supply in Drought‐Stressed Sorghum

Drought stress at flowering significantly altered functional group composition of the microbial community, with strong genotype‐specific effects (Figure 5a). The overall decline in bacterial abundance reflects the high sensitivity of these prokaryotic taxa to water limitation, impairing growth and eventually leading to cell shrinkage or death (Karagulyan et al. 2022; Ma et al. 2020). Gram‐negative bacteria, with thinner peptidoglycan layers, are particularly vulnerable, explaining their marked reduction under drought (Bouskill et al. 2025; Wan et al. 2023). An early increase in cyclopropane:precursor ratios at flowering indicates physiological stress of Gram‐negative bacteria (Zhu et al. 2023; Willers et al. 2015). Actinobacteria declined under drought for IESH and Gd but not for Mkl (Figure S4), indicating genotype‐specific root‐actinobacteria interactions, that affect robustness of these microbes (Naylor and Coleman‐Derr 2018; Xu et al. 2018; Ebrahimi‐Zarandi et al. 2023; Canarini et al. 2024). The sustained LAP enzymatic activity despite reduced prokaryotic abundance indicates a decoupling between microbial abundance and their functions in the sorghum rhizosphere under drought stress, suggesting a distinct shift to more drought‐resistant but highly active community members (Schimel 2018; Wan et al. 2023; Bouskill et al. 2025).

Under prolonged drought, however, the functional composition of the microbial community partially recovered, and the stress indicator ratio did not further increase (Table 2), suggesting that early drought‐induced physiological stress is followed by functional resilience of the sorghum's rhizosphere microbiome. The observation that specifically Actb2 and Actb3 recovered to levels comparable to moist soil at grain‐filling (Supporting Information Figure S4) agrees with the results of (Xu et al. 2018). They found that drought led to pronounced enrichment of antibacterial taxa in sorghum rhizosphere, which was attributed to the drought robustness of their filamentous structure and their ability to utilise recalcitrant C sources in nutrient‐poor environments (Ebrahimi‐Zarandi et al. 2023; Naylor and Coleman‐Derr 2018). This suggests that even in our nutrient poor tropical soil, actinobacteria adapt to drought by either exploiting alternative organic substrates or by direct interaction with an altered root exudate profiles under stress.

Among all microbial groups, fungal PLFA abundance was most resistant to drought and remained unchanged across genotypes. Their abundance did not reduce at flowering stage resulting in a higher fungal:bacterial ratio, pointing towards the reliance on the resistance of the fungal hyphal network to cope with initial stress (Canarini et al. 2024; Gao et al. 2021). Only later, under grain filling, when actinobacteria had recovered, the fungal:bacterial ratio was reduced suggesting a shift towards rapid, bacteria‐driven nutrient turnover (Jiang et al. 2025;). This transition is not indiscriminate (Figure 5b), that is, under drought, the sorghum plants selectively promoted symbiotic AMF (16:1ω5c), while the saprotrophic fungi (18:2ω6,9) was more associated with well‐watered conditions. This pattern suggests that sorghum strategically maintains symbiosis with AMF under drought to potentially enhance direct nutrient acquisition via mycorrhiza (Munene et al. 2025), while relying on bacterial partners to sustain mineralisation (Jiang et al. 2025; Bortolot et al. 2024). Taken together, we observed highly coordinated shifts in rhizosphere microbial composition and related functions that secure N acquisition during reproductive growth under drought (Boudabbous et al. 2022; Tang et al. 2022).

### 4.5 Role of AMF and Associated Microbes in Sustaining N Acquisition Under Drought

The association of the plants' N content and LAP enzyme activity with diverse bacterial taxa under optimal water supply at flowering (Figure 6a) suggests that a number of microbial groups are jointly involved in nutrient mineralisation and cycling, when resources are highly accessible (Qu et al. 2023; Bogati and Walczak 2022). One notable interrelation between composition of functional microbial groups and N acquisition functions under drought (Figure 6) was the positive association between fungal markers (AMF and saprotrophs) and chitinase activity.

This underlines the key role of the fungal‐associated pathways to chitin degradation specifically under water stress (Munene et al. 2025; Farhaoui et al. 2025). If direct N uptake is constrained by drying soils at this early reproductive stage, the establishment of fungal associations linked to nutrient cycling may function as a preparatory mechanism that helps buffer the plants against subsequent nutrient shortages. This functional shift in belowground partners, from bacteria facilitating fast N turnover under well‐watered conditions to fungi (AMF and saprotrophs) supporting nutrient release under drought, highlights the plasticity and strategic adaptation within the plant‐soil microbiome (Wu et al. 2025). This ideally exploits the high response dynamics and activities of prokaryotes together with the strong drought resistance of fungal hyphal networks, which are able to bridge air gaps and allocate water across dried macro‐pores.

By grain‐filling, the role of AMF in N allocation became more pronounced, coinciding with the enrichment of bacterial groups (e.g., Actinobacteria (10Me18:0) and Gram‐negative taxa (18:1ω7c) most likely in their hyphosphere. This pattern is consistent with mutualistic association that may contribute to sustaining nutrient availability and allocation to grains under stress (Zaman et al. 2024; Remke et al. 2021). The positive association between AMF and NU_t_E may reflect improved P acquisition (Jin et al. 2024), which can support photosynthesis. This supports C assimilation, delays the onset of senescence, and thereby prolongs the grain‐filling phase especially under drought condition (Yang et al. 2022; Li et al. 2017), all processes that may be of central importance for the sorghum plants in this experiment, too. While AMF are not direct producers of LAP, the channelling of plant‐derived C may stimulate AMF‐associated bacterial communities, potentially contributing to LAP production to cover hyphal and plant N demand (Jin et al. 2024). Actinobacteria, as K‐strategists, sustain nutrient turnover via slow decomposition of recalcitrant organic matter (Zhou et al. 2016), while Gram‐negative bacteria, often r‐strategists, rapidly exploit nutrient pulses from microbial and root exudate turnover (Yang et al. 2023; Cébron et al. 2021). This functional complementarity ensures N supply from both stable and transient N pools at this critical stage for yield formation.

Moreover, AMF was strongly associated to both ^15^N uptake and CANU under drought (Figure 6b; (Yang et al. 2022; Basiru et al. 2025), consistent with a potential role AMF‐associated networks in supporting plant N nutrition. Similar findings have been reported, with AMF enhancing post‐anthesis N uptake in maize, especially in nutrient‐deficient soils (Tian et al. 2023; Hui et al. 2022). This becomes especially important when nutrient availability is limited, as AMF hyphae access N pools in soil microsites beyond the reach of roots (Wang et al. 2024).

While AMF was positively associated with post‐anthesis ^15^N recovery, this relationship may reflect multiple interacting processes rather than direct fungal N transfer alone. AMF has been shown to improve plant water relations under drought by maintaining root hydraulic conductivity and water uptake, which may facilitate mass‐flow delivery of mineral N to roots independently of direct fungal N transport (Bitterlich et al. 2018; Abdalla and Ahmed 2021). In addition, AMF‐mediated C transfer may stimulate rhizosphere microbial activity and organic N mineralisation, thereby increasing N availability and potentially contributing to the observed association with ^15^N recovery. However, the compartmented experimental design used by Munene et al. (2025) demonstrates that all genotypes have the capacity for AMF‐mediated ^15^N‐uptake which still makes this process pretty plausible in contributing to the observed interrelation.

Together, these patterns suggest a shift from bacterial‐dominated nutrient release towards greater involvement of fungal‐associated processes during grain‐filling under drought is crucial for plant survival and productivity. The plant, AMF, and N‐mineralising bacteria act in functional complementary roles, improving nutrient use efficiency and resilience under drought stress in sorghum rhizosphere.

## 5 Conclusion

Drought significantly impacts N availability and thus acquisition and allocation in semi‐arid cropping systems. Our study highlights how reduced soil moisture shapes N acquisition strategies during reproductive stages among three sorghum genotypes (Figure 7).

**Figure 7:**
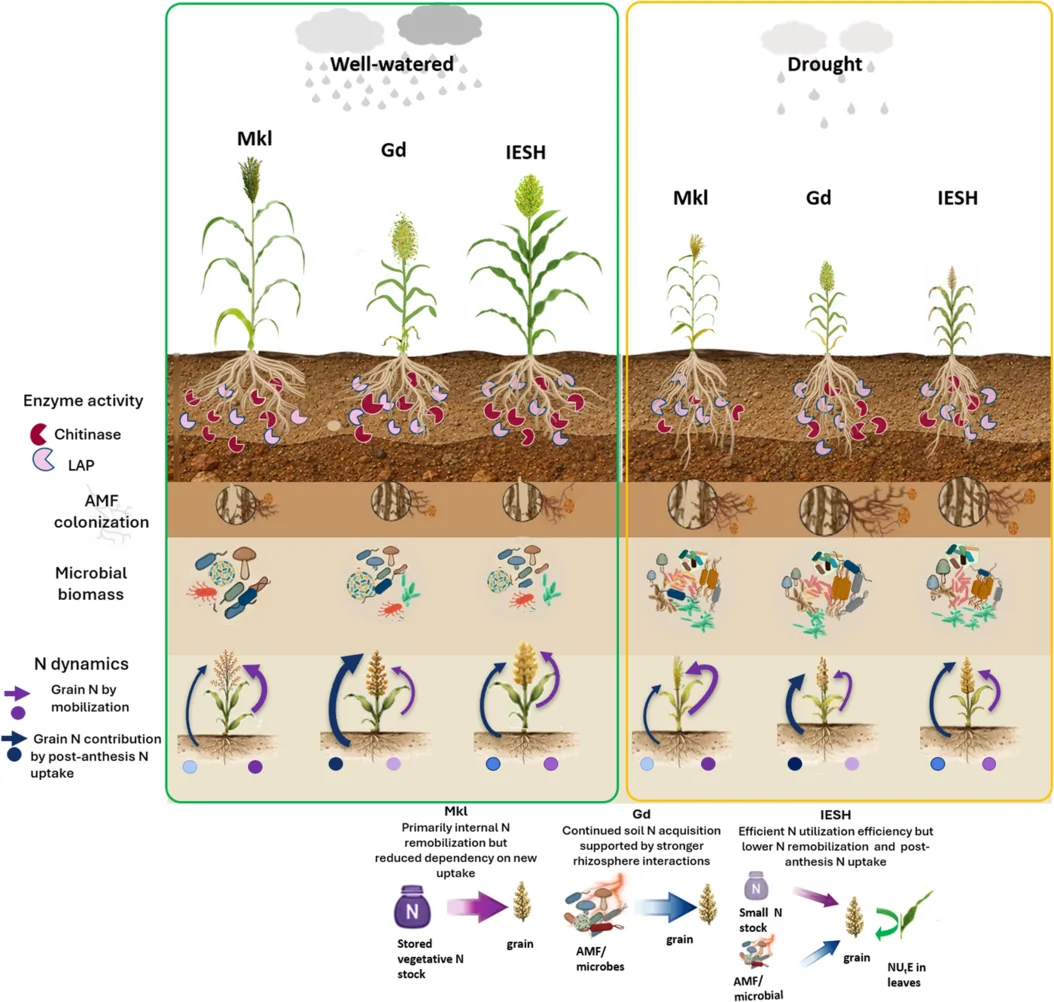
Schematic representation of nitrogen acquisition and allocation in three sorghum genotypes under well‐watered and drought conditions in sorghum genotypes (Makueni local; Mkl, Gadam; Gd and IESH 22012; IESH). The diagram illustrates shifts in microbial biomass, AMF colonisation, enzymatic activity, N utilisation efficiency (NU_t_E), post‐anthesis N uptake (dark blue), and N remobilisation efficiency purple). [Color figure can be viewed at wileyonlinelibrary.com]

The observed variation in adaptive diversity in N acquisition traits ranged from pre‐anthesis remobilisation in MKl to post‐anthesis uptake by Gd. Maintenance of mineralisation‐derived Nmin supply was associated with shifts in microbial enzymatic dynamics, from chitinase to LAP, enabling the exploitation of energetically less costly N sources under drought in the present study. AMF played a key role in facilitating post‐anthesis N acquisition and improving nutrient acquisition and utilisation efficiency (NU_t_E), particularly in genotypes with limited plant‐internal remobilisation capacity under stress such as Gd. Importantly, these benefits were reinforced through the recruitment of drought‐resilient Actinobacteria and Gram‐negative taxa by AMF which were linked to nutrient cycling and enzymatic activity. Together, these interactions helped sustain grain nutrient supply and maintain productivity under drought on this semi‐arid low‐fertile soil.

From a breeding perspective, within the genetic contrasts examined here, integrating physiological traits such as stay‐green linked drought resilience, nutrient remobilisation efficiency, and adaptive post‐anthesis uptake with root‐associated microbiome traits (e.g., AMF responsiveness, recruitment of a robust hyphosphere microbiome, enzymatic activity) offers a potential complementary multi‐mechanistic strategy. Such integration likely results in genotypes with greater functional synergy than others, relying on a single strategy, thereby enhancing nutrient acquisition and subsequent yield stability under drought stress.

## Conflicts of Interest

The authors declare no conflicts of interest.

## Supporting information


Supporting File

## Data Availability

The data that support the findings of this study are available from the corresponding author upon reasonable request.
