# Supplementary material for: Beneath the Drought: Rhizosphere Processes Shape Genotype‐Specific Sorghum Responses to Drought‐Induced N Limitation
Source: Plant Cell Environ. 2026 Aug 31;49(12):9752–69. doi: 10.1111/pce.70845 (PMC13725246; doi:10.1111/pce.70845)
Supplement: Supplementary file 1 [file PCE-49-9752-s001.docx]

# **Supporting information_Munene et al.,**

***Figure S1.*** *Shoot biomass (a) at flowering and at grain filling (b), grain yield (c), in sorghum genotypes under Well-watered (green) and drought (orange) conditions. Sorghum genotypes Mkl (Makueni local), Gd (Gadam), and IESH 22012. Different letters indicate significant differences (p<0.05) between treatments (water availability and genotypes) identified by* LMER*. p-values of ANOVA of water availability (W), genotype (G) and their interactions are indicated as ***p<0.001, **p<0.01, *p<0.05 and ns - not significant*

**Figure S2:** K_m_ values (µmol g^−1^ soil) at flowering; for chitinase (a), and leucine aminopeptidase; LAP (c) and at grain-filling; chitinase (b) and LAP (d) under well-watered (left) and drought (right) conditions. Soil depths: 0-30 cm; light brown inverted triangle, dark brown square; 30-60 cm. The sorghum genotypes: Mkl (Makueni local), Gd (Gadam), and IESH 22012. Data are presented as means ± SE. Different capitalized and small letters indicate significant differences (p < 0.05) between sorghum genotypes and water availability on 0-30 cm and 30-60 cm respectively. p-values of ANOVA of water availability (W), genotype (G) and their interactions are indicated as ***p<0.001, **p<0.01, *p<0.05 and ns - not significant

**Figure S3:** Chitinase:LAP ratios values (µmol g^−1^ soil) at flowering (a) and grain-filling (b) under well-watered (left) and drought (right) conditions. Soil depths: 0-30 cm; light brown inverted triangle, dark brown square; 30-60 cm. The sorghum genotypes: Mkl (Makueni local), Gd (Gadam), and IESH 22012. Data are presented as means ± SE. Different capitalized and small letters indicate significant differences (p < 0.05) between sorghum genotypes and water availability on 0-30 cm and 30-60 cm respectively. p-values of ANOVA of water availability (W), genotype (G) and their interactions are indicated as ***p<0.001, **p<0.01, *p<0.05 and ns - not significant

***Figure S4:*** *Relative abundance of microbial biomass groupings at flowering (left) and grain-filling(right) stagesunder well-watered (green) and drought (oraange) conditions****.*** *G+1 (i15:0, i16:0), G+2 (a15:0, i17:0), G+3 (a17:0) which are Gram positives, Actb 1 (10Me16:0), Actb 2 (10Me17:0), and Actb 3 (10Me18:0) as Actinobacteria, G-1 (16:1ω7c), G-2 (18:1ω7c) as Gram negatives, AMF (16:1ω5c), Fungi (18:2ω6,9) as saprotroph fungi, and cy17:0 and cy19:0 as stress markers* *Net The sorghum genotypes Mkl (Makueni local), Gd (Gadam), and IESH 22012. Different letters indicate significant differences (p<0.05) between treatments (water availability genotypes). p-values of ANOVA of water availability (W), genotype (G) and their interactions are indicated as ***p<0.001, **p<0.01, *p<0.05 and ns - not significant*

***Figure S5:*** *Net Assimilation (a-b), transpiration (c-d) and stomata conductance (e-f) at/during flowering and grain-filling stages respectively for the three sorghum genotypes under well-watered (green) and drought (orange) conditions. The sorghum genotypes Mkl (Makueni local), Gd (Gadam), and IESH 22012. Different letters indicate significant differences (p<0.05) between treatments (water availability genotypes). p-values of ANOVA of water availability (W), genotype (G) and their interactions are indicated as ***p<0.001, **p<0.01, *p<0.05 and ns - not significant*

***Figure S6: .*** *Soil moisture content (SMC) at 0-30 cm (solid) and 30-60 cm (dashed) depths from weeks 5-15 under well-watered ( blue) and drought (brown) conditions.*

***Figure S7.*** *Root distribution, expressed as root counts determined by profile-wall root mapping ("root painting"; in situ tracing of visible roots onto transparent polythene sheets placed against the exposed soil profile), at 0-30 cm (light brown) and 30-60 cm (dark brown) soil depths under well-watered and drought conditions across the three sorghum genotypes (Mkl, Gd and IESH) at flowering (a) and grain filling (b). Box plots show the median, interquartile range, whiskers, and outliers. Different letters indicate significant differences (p < 0.05) among genotype × water treatment combinations based on linear mixed-effects models (LMM). Significance levels are indicated as ***p < 0.001, **p < 0.01, *p < 0.05, and ns = not significant.*

**Table S1: Grouping of the PLFAs to microbial groups: twelve groups were distinguished through factor analysis and relevant factor loadings are marked as red and bold.**

| Fatty acid | Factor 1 | Factor 2 | Factor 3 | Factor 4 | Factor 5 | Microbial group |
| --- | --- | --- | --- | --- | --- | --- |
| i15:0 | -0.462 | -0.383 | -0. 415 | **0.741** | 0.042 | Gram-positives 1  (Gram+1) |
| i16:0 | -0.052 | 0.055 | 0.024 | **0.957** | 0.0002 |  |
| a15:0 | 0.062 | **0.930** | -0.038 | 0.111 | -0.023 | Gram-positives 2  (Gram+2) |
| i17:0 | -0.379 | **0.637** | 0.110 | 0.029 | 0.014 |  |
| a17:0 | **0.927** | -0.050 | 0.116 | -0.015 | 0.162 | Gram-positives 3 (Gram+3) |
| 16:1w7c | 0.067 | **0.787** | 0.011 | 0.115 | -0.298 | Gram-negatives 1  (Gram-1) |
| 18:1w7c | 0.087 | 0.219 | 0.147 | **0.678** | -0.533 | Gram-negatives 2  (Gram-2) |
| 16:1w5c | **0.868** | 0.072 | -0.110 | -0.007 | -0.332 | AMF |
| 10Me16:0 | -0.031 | 0.306 | -0.174 | -0.444 | **0.556** | Actinobacteria 1  (Actb1) |
| 10Me17:0 | 0.151 | 0.014 | **0.831** | 0.236 | -0.136 | Actinobacteria 2  (Actb2) |
| 10Me18:0 | **0.907** | -0.012 | -0.016 | -0.015 | 0.055 | Actinobacteria 3  (Actb3) |
| 18:2w6,9 | -0.135 | -0.035 | **0.863** | -0.325 | 0.127 | Saprophytic fungi |
| cy17:0 | 0.036 | 0.157 | **0.814** | 0.196 | -0.070 | Stress indicator 1 (for G-1) |
| cy19:0 | **-0.698** | -0.066 | -0.266 | -0.282 | 0.082 | Stress indicator 2 (G-2) |

***Figure S8:*** *Treatment-level responses of grain N content (a) and leucine aminopeptidase (LAP) activity at flowering (b) and grain filling (c). Data are pooled across the three sorghum genotypes and presented as boxplots comparing well-watered(green) and drought (orange) treatments.*
